# 4222 Synthesis and application of cyanuric chloride lipids for peptide presentation

**DOI:** 10.1017/cts.2020.94

**Published:** 2020-07-29

**Authors:** David Nardo, Vincent J. Venditto

**Affiliations:** 1 University of Kentucky Center for Clinical and Translational Science; 2 University of Kentucky

## Abstract

OBJECTIVES/GOALS: My long-term career objective is to become an established independent researcher focused on understanding and modulating immune responses to biologics in order to enhance their efficacy and understand the underlying mechanisms by which these interact with the immune system. METHODS/STUDY POPULATION: In this study we will evaluate the utility of cyanuric chloride based synthetic lipids in the presentation of peptide epitopes of the gene delivery vector, adeno-associated virus (AAV). The lipopeptide conjugates will be administered to mice via liposomal formulations to assess their ability to induce immune responses by ELISA as compared to mice treated with the AAV. The three-dimensional conformation of the peptides will be evaluated using nuclear magnetic resonance to determine their similarity with the natural conformation that these peptides adapt on the viral surface. Additionally, to assess the translatability of this conjugation strategy, we will test the ability of our lipopeptides to bind to serum antibodies from human subjects. RESULTS/ANTICIPATED RESULTS: We anticipate that peptide presentation using cyanuric chloride lipids will achieve a robust response in mice following immunization. Immunizations with our lipids should induce the production of antibodies targeting AAV, albeit a milder response that the virus itself, given the complexity of viral epitopes. Nuclear magnetic resonance will inform us on how to improve the synthetic lipids to optimize peptide presentation by altering the characteristics of the lipid anchors. Finally, by using human serum to test for the ability of our lipopeptides to bind to antibodies in serum from patients positive for AAV antibodies, we can become informed on whether our strategy has utility in human studies or whether our method is limited to animal models of human disease. DISCUSSION/SIGNIFICANCE OF IMPACT: The current work seeks to develop a strategy to present peptides from a well characterized biologic, AAV, on a liposome surface. Our ultimate purpose is to employ liposomal formulations as decoys that target AAV-specific lymphocytes to improve the *in vivo* efficacy of AAV.

